# Surgical outcomes following resection in patients with language dominant posterior quadrant epilepsy

**DOI:** 10.1016/j.ebr.2024.100695

**Published:** 2024-07-15

**Authors:** Satvir Saggi, Joseph H. Garcia, Faraz Behzadi, Arka N. Mallela, Paul A. Garcia, Edward F. Chang, Robert C. Knowlton

**Affiliations:** a Department of Neurological Surgery, University of California San Francisco, San Francisco, CA, USA; b Department of Neurological Surgery, University of Pittsburgh Medical Center, Pittsburgh, PA, USA; c Department of Neurology, University of California San Francisco, San Francisco, CA, USA; d Department of Radiology, University of California San Francisco, San Francisco, CA, USA

**Keywords:** Epilepsy, Posterior Cortex, Refractory, Resection

## Abstract

Posterior quadrant epilepsy surgery, involving the occipital lobe, parietal lobe, or the posterior border of the temporal lobe, accounts for a small percentage of focal resections for medically refractory epilepsy. Prior studies investigating seizure control from posterior quadrant epilepsy surgery are limited. In this study, a retrospective database of patients undergoing surgery for left sided posterior cortex epilepsy at a single large level 4 epilepsy center was analyzed between August 2008 to April 2021 in order to characterize seizure control outcomes. Nine patients presented with epileptogenic foci in the left posterior cortex with a malformation of cortical development deemed as the etiology of seizures for all but one patient. Absolute seizure freedom (Engel I) was achieved in 4 of 9 patients, with the remaining 5 patients achieving an improvement in the frequency of seizures (Engel II/III). Complete resection of the anatomic and physiologic abnormalities was performed in 3 of 4 patients with Engel 1 outcomes and 1 of 5 patients with Class II/III outcomes. Five patients developed new right sided visual field defects, all of which were expected based on the sub-lobar, occipital localization and were viewed as acceptable by the patients and did not interfere with activities of daily living. Overall, our study demonstrates the potential for surgical resection to yield excellent seizure-control outcomes with anticipated, tolerable neurological deficits. This information is important for patients with disabling seizures who may not benefit sufficiently from palliative procedures.

## Highlights

• Studies investigating seizure control from posterior quadrant epilepsy surgery are limited.
• Patients with complete resection led to better outcomes, though all improved.
• Surgery can yield excellent seizure-control outcomes with anticipated, tolerable deficits.

## 1 Introduction

Posterior quadrant epilepsy surgery, involving the occipital lobe, parietal lobe, or the posterior border of the temporal lobe, accounts for only 5–10 % of focal resections for medically refractory epilepsy [1], [2]. The low frequency of this form of epilepsy and the difficulty in localization of epileptogenic foci make surgical treatment for seizures localized to the posterior cortex relatively rare compared to resection for seizures from the anterior temporal and frontal cortical regions [3], [4]. Clear anatomic or neurophysiologic distinctions are challenging to identify among these areas, and the epileptogenic regions are not always limited within the borders of the occipital, parietal, or posterior temporal lobes.

Numerous functions are localized to the posterior cortical regions. The parietal lobe integrates sensation and cognition, and acts as a sensorimotor interface [4]. The occipital cortex functions mainly as the center for the recognition, integration, and processing of visual perceptions [5]. The complexity of posterior cortical function contributes to the difficulty of surgical planning and evaluating changes following surgery [4], [5].

Factors influencing language localization have theoretical importance in the understanding of the organization and reorganization of higher cognitive functions, as well as having practical implications in neurosurgery and subsequent rehabilitation. Atypical language representation is more frequent in focal epilepsy than in healthy people. This difference is thought to be related to early childhood brain adjacent to language centers [6], [7].

Although predictors of favorable outcomes related to seizure control and postoperative language deficits in temporal lobe epilepsy are well established, comparable studies of favorable seizure control in the posterior quadrant have been less clear cut [2], [3], [8]. Notably, prior predictive studies of seizure control from posterior quadrant epilepsy surgery have taken place in small cohorts and have rarely utilized language mapping or source localization tools [3], [4]. Given the current paucity of data in this realm, our team sought to characterize surgical approaches and outcomes in a cohort of patients with language dominant posterior epilepsy.

## 2 Methods

A retrospectively generated database of patients undergoing surgery for left sided posterior quadrant epilepsy at a single large level 4 epilepsy center was analyzed between August 2008 and April 2021. Patients were extracted from a larger list of patients with seizures arising from the posterior cortex. The clinical assessment of symptomatic presentation was based on assessment by a neurologist. The initial patient presentation was defined as the clinical event that led to initial presentation and subsequent diagnosis of epilepsy. Semiologies for presenting patients were characterized in detail by epileptologists.

All patients included underwent further evaluation with MRI, EEG, and electrical source localization. A subset of patients underwent additional studies including, 2-[^18^F]fluoro-2-deoxy-D-glucose positron emission tomography (FDG-PET), magnetoencephalography (MEG), and ictal single photon emission computed tomography (iSPECT). These non-invasive tests were used to tailor placement of intracranial electrodes for each patient rather than using standard electrode placements. All imaging and neurophysiological abnormalities were included in the coverage. Subdural grids were typically used in regions where language mapping was critical, while depth electrodes were utilized to sample tissue inaccessible to subdural electrodes (Table 1). Depth electrodes were used to record from the hippocampus when imaging supported the possibility of dual pathology (hippocampal atrophy or increased signal) and medial temporal structures were resected if included in the ictal onset zone. Functional mapping by electrical cortical stimulation included language (naming, repetition and reading) if the seizure onset zone was near classical language areas. Visual and somatosensory responses were obtained when necessary to tailor the resection.

**Table 1:** Characteristics of 9 patients with epileptogenic foci in the left posterior cortex.

**Case**	**Age at Surgery**	**Age at Onset of Seizures**	**Handedness**	**Sex**	**Ethnicity**	**Pre-operative MRI Findings**	**Co-existing Hippocampal MRI Abnormalities**	**Post-operative Pathology Finding**	**Intracranial Electrodes**	**Years Follow Up From Surgery**
1	12	7	Right	Female	Caucasian	MCD	−	MCD	SD grids and strips	12
2	4	3	Right	Female	Caucasian	MCD	+	MCD	SD grids and strips	2
3	33	28	Right	Male	Caucasian	MRI-negative	−	MCD	SD grids and strips + depths	6
4	33	3	Right	Female	Hispanic	Gliosis	+	Gliosis	SD grids and strips + depths	9
5	22	3	Right	Female	Caucasian	MCD	−	MCD	SD grids and strips + depths	1
6	34	2	Right	Female	Caucasian	Gliosis	−	MCD	SD grids and strips + depths	6
7	39	2	Right	Female	Caucasian	MRI-negative	−	MCD	SD grids and strips + depths	7
8	32	13	Right	Male	Hispanic	MCD	−	MCD	SEEG	8
9	25	23	Right	Male	Other	MCD	+	MCD	SD grids and strips + depths	2

The decision to offer resection (as opposed to no surgical treatment or isolated neurostimulation) was only undertaken when the risk of ongoing seizures, in the judgment of the patient (or guardian) and our multidisciplinary team, outweighed the expected deficits. The surgical goal was for complete resection of the structural and neurophysiological abnormality, whenever possible and the approach to tailoring the resection based on imaging as well as ictal and interictal recordings has been published previously [9]. In this group of patients with severe, disabling seizures, resection of language-related functional tissue was typically avoided, while primary visual cortex was sometimes sacrificed after extensive discussion with the patient.

Variables related to age, sex, and ethnicity were recorded retrospectively through analysis of patient charts. Antiepileptic drugs were initiated and managed by neurologists and were also obtained through retrospective chart review. Seizure classification was based on clinical features described in the medical records. Additionally, Engel classification scores were assigned to patients at each follow-up by epileptologists following their cases [10].

## 3 Results

Nine patients presented with epileptogenic foci in the left posterior quadrant (Table 1) with an average follow up of 5.9 years following surgery. Of these 9 patients, 6 were female. Average age at onset of seizure activity was 9.3 years with an average age at surgical resection of 27.3 years. 7 of 9 patients demonstrated pre-operative MRI abnormalities with a MCD found to be the most common MRI abnormality. Following post-operative pathology of resected tissue, a MCD was deemed as the etiology of seizures for all but one patient. Absolute seizure freedom (Engel Class I) was achieved in 4 of 9 patients, with the remaining 5 patients achieving an improvement in the frequency of seizures (Engel Class II/III). All of the patients with residual seizures have had remarkable improvements in seizure control. Case 3 and Case 7, who presented with MRI-negative findings and subsequently found to have a MCD on post-operative pathology, were both found to have incomplete seizure remission (Engel Class III) following surgery. However, both patients have not had seizures for the most recent year of last follow-up. Patients 1 and 8 have infrequent, exclusively nocturnal seizures and patient 4 has seizures about once per year, except with medication lapses. Complete resection of the anatomic and physiologic abnormalities was performed in three of four patients with Engel 1 outcomes and 1 of 5 patients with Class II-III outcomes. The residual epileptogenic tissue is listed in Table 4. Despite a large temporo-parietal resection, Patient 4 had residual, continuous discharges recorded in Classical Wernicke’s area and elected to have a responsive stimulator placed over that region in addition to the resection.

Functional mapping was performed in seven of nine patients with 5 patients demonstrating overlap of epileptogenic foci with eloquent cortex as determined by functional mapping or based on classical anatomy (Table 2; Table 3). Of the two not mapped, Case 2 was only 4 years old and Case 1 had surgery with the expectation of new visual deficits, making mapping superfluous. Specifically, visual responses were identified in Case 3 and 8, while reading disruption was identified in patient 7. Intracranial EEG indicated that the seizure focus included occipital cortex in seven patients, temporal cortex in eight patients, and parietal cortex in two patients. Only patients 3 and 5 had seizure onsets confined to a single lobe.

**Table 2:** Electrical stimulation mapping results ^a.^

**Function**	**Proportion of Patients with Positive Mapping Result**	**Case Numbers with Positive Results**
Language comprehension (Wernicke’s)	0/7	N/A
Reading	1/7	7
Writing	0/7	N/A
Recognition of objects/people (Agnosia)	0/7	N/A
Vision	2/7	3, 8

^a^Electrical stimulation mapping was not performed in 2 patients (Case 1 and Case 2).

**Table 3:** Epilepsy localization and post-operative outcomes.

**Case**	**Occipital Epilepsy Localization**	**Temporal Epilepsy Localization**	**Parietal Epilepsy Localization**	Overlap of Epileptogenic Foci with Eloquent Cortex *	**Deficits Before Surgery**	**Deficits After Surgery**	**Engel Class**
1	LOi, LOs, BO, MO	BT	None	+	None	Right homonymous hemianopsia, reading difficulty	Class III
2	LOi, LOs, BO, MO	LT, TP, BT, MT	None	−	Receptive/expressive aphasia	Right homonymous hemianopsia, receptive/expressive aphasia (improved)	Class I
3	LOi	None	None	+	None	Right homonymous superior quadrantopia	Class III
4	None	OpT	APi	+	None	None	Class II
5	None	BT	None	−	None	None	Class I
6	LOi, LOs, BO, MO	BT, LT	PPs, PPi, APs	+	Visual field deficit	Visual field deficit (stable)	Class I
7	BO	BT	None	−	Auditory deficits, confrontational naming	Right homonymous superior quadrantopia	Class III
8	BO	BT	None	+	None	Reading difficulty	Class III
9	MO	MT, BT, TP	None	−	None	Right homonymous superior quadrantopia, expressive aphasia	Class I

*LOi* Lateral occipital inferior; *LOs* Lateral occipital superior; *BO* Basal occipital, *MO* Medial occipital; *LT* Lateral temporal; *TP* Temporal polar; *BT* Basal temporal; *MT* Medial temporal; *OpT* Operculum temporal; *APi* Anterior parietal inferior; *APs* Anterior parietal superior; *PPi* Posterior parietal inferior; *PPs* Posterior parietal superior.

*Eloquent cortex was determined based on functional mapping by electrical cortical stimulation or by classical anatomy.

**Table 4:** Location of Resected and Incompletely Resected Epilepsy Localization Areas.

Case	Completely Removed Epileptiform Tissue	Incompletely Removed Epileptiform Tissue
1	LOi, LOs, BO, MO	BT**
2	LOi, LOs, BO, MO, LT, TP, BT, MT	None
3	None	LOi*
4	APi	OpT**
5	BT	None
6	LOi, LOs, BO, MO, BT, LT, PPs, PPi	Aps**
7	BO, BT	None
8	None	Incomplete resection of band heterotopia**
9	MO, MT, BT, TP	None

*LOi* Lateral occipital inferior; *LOs* Lateral occipital superior; *BO* Basal occipital, *MO* Medial occipital; *LT* Lateral temporal; *TP* Temporal polar; *BT* Basal temporal; *MT* Medial temporal; *OpT* Operculum temporal; *APi* Anterior parietal inferior; *APs* Anterior parietal superior; *PPi* Posterior parietal inferior; *PPs* Posterior parietal superior.

*Patient 3 was found to have eloquent tissue in this area based on functional mapping by electrical cortical stimulation and therefore, did not have tissue resected from this area.

*Patients 1, 4, 6, and 8 and were believed to have eloquent tissue in these areas based on classical anatomy and consequently, did not have tissue resected from these areas.

Three patients presented with preoperative neurologic deficits. Case 2 presented with receptive/expressive aphasia which was noted to be relatively improved post-operatively. However, the patient developed a new right homonymous hemianopsia post-operatively. Case 6 presented with general visual field deficits preoperatively which remained stable following surgery. Case 7 presented with auditory deficits and an inability to perform confrontational naming of which both resolved following surgery. Four patients presented without preoperative neurologic deficits but subsequently developed a new postoperative neurologic deficit. Cases 1, 2, and 9 all developed new postoperative right visual field defects with cases 1 and 9 additionally developing new deficits in reading ability and transient expressive aphasia, respectively. Case 8, as with case 1, developed a new deficit in reading ability. Finally, Case 3 and 7, both of which were found to have a MCD following post-operative pathology, developed a right homonymous superior quadrantopia following surgery.

## 4 Discussion

The nine patients studied in this case series were predominantly female (66.7 %) with the majority presenting at an age of onset of seizures younger than 18 (77.8 %). Although the extent of prior studies on posterior quadrant epilepsy and their treatment is limited, our patient population’s age and sex distribution are comparable to the literature [1], [3]. Our patient population is likely not fully representative of all patients with refractory, dominant, posterior focal epilepsy. The decision to offer resection was only undertaken when the risk of ongoing seizures outweighed the expected deficits. Furthermore, only patient 4 was treated with responsive neurostimulation and then only after it became clear that further resection had a high chance of causing aphasia. Similar to previous literature, many patients had inconclusive brain mapping for the expected deficit prior to treatment [11].though the functional outcomes were expected based on classical anatomy.

All patients with no apparent findings on pre-operative MRIs (cases 3 and 7) were observed to have an Engel class III outcome post-resection. MRI-negative, focal epilepsy is associated with less successful surgical outcomes based on the lack of a pathophysiologic understanding of the illness. Additionally, such patients often fail to meet the criteria to be included in surgical studies, limiting investigation into this form of epilepsy [12]. Three of the four Engel class III patients (75 %: Cases 3, 7, 8) had functional tissue identified during brain mapping, two of which (50 %: Cases 3 and 7) had MRI-negative scans. Our extensive series of patients undergoing surgical treatment of cortical malformations demonstrated that incomplete resection of the anatomic or ECoG abnormality was associated with residual seizures [9]. That pattern held in all but two of the patients in this series. Patient 6 became seizure-free despite discharges in residual superior parietal tissue and Patient 7 had residual seizures despite having a complete resection.

Six of the patients had new neurologic deficits from pre-op to post-op (Cases 1, 2, 3, 7, 8, 9). Of the six patients, one (Case 3) had evidence of a positive pre-operative functional mapping result for the related deficit. Three patients (Cases 4, 5, 6) remained stable with no new neurologic deficits pre-op to post-op, and one patient (Case 2) experienced an improvement in their neurological deficit pre-op to post-op.

According to prior studies, a higher number of localized radiographic or electroencephalographic epileptogenic foci does not necessarily correlate with the chances of seizure freedom post-operatively, however, the specific location of the lesion may [13], [14], [15]. For example, most patients in this study with epileptogenic foci in the temporal lobe with a localization specifically present in the basal temporal lobe did not experience full seizure remission (Engel Class III; Cases 1, 7, 8). Similarly, patients with epileptogenic foci in the occipital lobe with a localization specifically present in the basal occipital lobe did not experience full seizure remission (Engel Class III; Cases 7, 8). Basal occipital epilepsies are rare and are described to be associated with visual auras and basilar migraines pre-operatively, and visual deficits post-operatively in nearly 20 % of cases as evidenced by Case 7 [16]. Presence or absence of localization in the parietal region had little apparent relationship with the seizure treatment outcomes or post-operative complications.

The rate of Engel class I outcomes in our study was 4/9 (44 %) which falls into the range of seizure freedom rates (42.1 % − 86.3 %) reported in a prior *meta*-analysis by Harward et. al for studies conducted in a similar time frame as this study [17]. The majority of post-operative visual complications (five of nine) were right sided field defects, all of which were expected based on the sub-lobar, occipital localization. The rate of visual complications in this series, is similar to other studies on posterior quadrant epilepsy surgical outcomes [17]. The rate of visual deficit in occipital epilepsy post-operatively was five out of seven (71 %) in our study which was similar to the rate (∼60–80 %) reported in literature [17], [5]. Importantly, these visual deficits were viewed as acceptable by the patients and overall, did not interfere with activities of daily living. Therefore, our small sample of patients with severe, posterior quadrant epilepsy suggests that patients and families must be counseled about the possibility of new visual deficits but they also need to consider this risk in the context of the good chance for meaningful seizure improvement. Similarly, with respect to language, no patients in our cohort developed lasting, disabling neurologic language deficits with respect to both expressive and receptive language abilities and were able to communicate without significant apparent deficits in their speech.

The onset age of epilepsy and duration of epilepsy did not show an apparent association with seizure freedom. Frontal cortex epilepsy literature has shown significantly better surgical outcomes in patients with longer duration of epilepsy and younger age of onset, but this property has not been replicated in posterior cortex literature [18], [19]. In most children with posterior epilepsy, the dysfunctional epileptogenic zone is resistant to medical therapy [20]. Therefore, the most appropriate treatment for these patients is surgical resection, and the literature suggests early surgical treatment may have better outcomes [20]. Our posterior quadrant epilepsy patients had an average time of onset to surgical treatment of 14.8 years. Nonetheless, we believe that more patient preference surveys and studies are needed to understand the reasoning behind later onset of surgical treatment in this vulnerable patient population.

Of final importance is the concept of dual pathology, defined as the presence of two epileptogenic lesions (e.g. hippocampal and extrahippocampal) with prior reports demonstrating the need for resection of mesial temporal lesions and extrahippocampal lesions [21]. In this study, three patients (Cases 2, 4, and 9) presented with co-existing hippocampal abnormalities on MRI with Case 2 and Case 9 demonstrating seizure onset zone including the mesial temporal lobe (Table 1, Table 3). Following surgical resection that included the mesial temporal lobe epileptiform tissue, both patients demonstrated Engel Class I outcomes, further demonstrating the importance of resection of mesial temporal lobe tissue in patients with dual pathology.

### 4.1 Limitations

This retrospective study is a case series limited to nine patients with severe, epilepsy who opted for aggressive, resective surgery knowing of the high likelihood of new deficits. Thus, it may not be generalizable to all patients with refractory, language-dominant, posterior epilepsy. However, given the paucity of literature on left sided posterior quadrant epilepsy, this study presents data for further surgical improvement in this understudied patient population.

## 5 Conclusion

Our study of surgical outcomes on nine language-dominant medically refractory posterior quadrant epilepsy patients demonstrates the potential for surgical resection to yield excellent seizure-control outcomes with anticipated, tolerable neurological deficits. This information is important for patients with the most disabling seizures who may not benefit sufficiently from palliative procedures. Complete resection of epileptogenic tissue, whenever possible, is associated with the best chance for seizure control.

## Ethical statement

This study has been performed in compliance with relevant laws and institutional guidelines and have been approved by the appropriate institutional committee.

## CRediT authorship contribution statement

**Satvir Saggi:** Writing – review & editing, Writing – original draft, Methodology, Formal analysis, Conceptualization. **Joseph H. Garcia:** Writing – review & editing, Writing – original draft, Methodology, Formal analysis, Data curation, Conceptualization. **Faraz Behzadi:** Writing – review & editing, Writing – original draft, Methodology, Investigation, Formal analysis. **Arka N. Mallela:** Writing – review & editing, Writing – original draft, Conceptualization. **Paul A. Garcia:** . **Edward F. Chang:** Writing – review & editing, Writing – original draft, Supervision, Project administration, Conceptualization. **Robert C. Knowlton:** Writing – review & editing, Writing – original draft, Validation, Supervision, Project administration, Formal analysis, Conceptualization.

## Declaration of competing interest

The authors declare that they have no known competing financial interests or personal relationships that could have appeared to influence the work reported in this paper.
